# Characterizing the Diffusion Property of Hydrogen Sorption and Desorption Processes in Several Spherical-Shaped Polymers

**DOI:** 10.3390/polym14071468

**Published:** 2022-04-04

**Authors:** Jae-Kap Jung, Kyu-Tae Kim, Nak-Kwan Chung, Un-Bong Baek, Seung-Hoon Nahm

**Affiliations:** 1 Hydrogen Energy Materials Research Center, Korea Research Institute of Standards and Science, Daejeon 34113, Korea; nk.chung@kriss.re.kr (N.-K.C.); ubbaek@kriss.re.kr (U.-B.B.); shnahm@kriss.re.kr (S.-H.N.); 2 Electricity and Magnetism Group, Korea Research Institute of Standards and Science, Daejeon 34113, Korea; ktkim@kriss.re.kr

**Keywords:** polymer, volumetric analysis, permeation, diffusion, hydrogen sorption, desorption

## Abstract

We developed a method for characterizing permeation parameters in hydrogen sorption and desorption processes in polymers using the volumetric measurement technique. The technique was utilized for three polymers: nitrile butadiene rubber (NBR), ethylene propylene diene monomer (EPDM), and fluoroelastomer (FKM). The total uptake ($C_{\infty }$), total desorbed content ($C_{0})$, diffusivity in sorption (D_s_), and diffusivity in desorption (D_d_) of hydrogen in the polymers were determined versus the sample diameter used in both processes. For all the polymers, the diameter dependence was not detected for $C_{\infty }$ and $C_{0}$. The average $C_{\infty }$ and $C_{0}$ at 5.75 MPa were 316 wt∙ppm and 291 wt∙ppm for NBR, 270 wt∙ppm and 279 wt∙ppm for EPDM, and 102 wt∙ppm and 93 wt∙ppm for FKM. The coincidence of $C_{\infty }$ and $C_{0}$ in the sorption and desorption process indicated physisorption upon introducing hydrogen molecules into the polymers. The larger D_d_ in the desorption process than D_s_ could be attributed to an increased amorphous phase and volume swelling after decompression. The equilibrium time to reach the saturation of the hydrogen content in both processes was experimentally confirmed as proportional to the squared radius and consistent with the COMSOL simulation. This method could be used to predict the equilibrium time of the sorption time, depending on the radius of the polymers without any measurement.

## 1. Introduction

Amorphous polymers and polymer-based amorphous composites are highly popular for conceiving desired functional applications in many fields [1,2,3]. Especially, the sorption and desorption of hydrogen into/from polymer membranes are very important processes controlling the permeation property and clarifying the fracture mechanism in the gas sealing applications of O-rings [4,5,6,7,8]. In particular, permeation effectiveness is associated with not only the equilibrium condition but also the dynamics of both processes under high-pressure environments [9,10]. Therefore, an investigation of the saturated equilibrium and sorption/desorption properties of hydrogen permeation is essential for designing polymer testing equipment, reducing operating costs, gaining insights into sorption, and, finally, determining the appropriate exposure time to hydrogen in cycling testing [11,12].

Previous studies have reported that hydrogen sorption mainly takes place in the amorphous phase in polymers [13,14]. Rapid decompression within a few seconds after hydrogen sorption at high pressure causes expanded hydrogen voids, thus allowing a change in the crystalline and amorphous phases. The morphological changes lead to differences in the permeation parameters between the sorption and desorption processes; moreover, the amorphous phase and free volume in the polymer work as the hydrogen diffusion path for hydrogen [14,15,16].

For comparison with a previous investigation and a finding of the sorption/desorption mechanism of H_2_, we developed a process for measuring the H_2_ sorption property during pressurization. In this process, the sorption property versus the variation in the residence time when exposed to high pressure, as well as the desorption property during decompression, were measured via a developed volumetric analysis technique (VAT) [17,18]. This work was conducted for rubbery polymers, nitrile butadiene rubber (NBR), ethylene propylene diene monomer (EPDM), and fluoroelastomer (FKM) utilized as the seal components for O-rings in H_2_ gas applications [19]. The total uptake ($C_{\infty }$), total desorbed content ($C_{0}$), diffusivity in sorption (D_s_), and diffusivity in desorption (D_d_) of H_2_ in the three polymers were determined versus the sample diameter in both processes. The generalized findings regarding the sorption and desorption parameters of H_2_, given the H_2_ content and diffusivity, were drawn from the experimental investigation of polymeric materials. In addition, we discuss herein the reversibility between the H_2_ sorption and desorption processes. Hysteresis in the diffusion observed for the two processes was examined in terms of the amorphous phase portion.

Moreover, the sorption and desorption equilibrium time at which the H_2_ content is saturated is very important for determining the high pressure (HP)-exposed conditions in cycling tests of polymers and for designing the material for HP H_2_ gas seal devices. The time to reach the sorption and desorption equilibrium was found to be dependent on both the specimen volume and diffusivity. The main concern is that the linear correlation between the equilibrium time and squared radius of the specimen is maintained, even though the diffusivity is not constant. If the linearity is true, then a prediction of the sorption equilibrium time required to design the O-ring seal could be possible. The experimental results of the diffusion properties of the two processes and equilibrium time were applied to research the HP H_2_ effect and to determine the exposure time of the hydrogen cycling test. The experimental investigations were compared and confirmed via finite element simulations using COMSOL.

## 2. Measuring System and Data Analysis

The functions and compositions of the polymers used are summarized in Table 1. The equipment used for the preparation of the polymer mixtures were two roll mills, a rheometer, and an oil hydraulic press. The curing conditions of the specimen were 170 °C and 10 min. The optimal vulcanization time and temperature by the rheometer were 180 s and 170 °C for NBR, 360 s and 170 °C for EPDM, and 300 s and 170 °C for FKM.

NBR was employed as the O-ring seal for the flange connection, threaded connector, and various valves in the high-pressure H_2_ refueling station because of its excellent gas resistance [20]. Meanwhile, EPDM is a synthetic rubber and has outstanding heat, weathering, and aging resistance [20]. EPDM exhibits excellent electrical insulation and low-temperature property but only fair physical strength property. It can be employed in a wide range of applications, such as in radiators, heater hoses, door seals, O-rings and gaskets, accumulator bladders, cable connectors and insulators, diaphragms, and weather stripping. FKM is a fluorocarbon-based synthetic polymer fabricated by copolymerizing tetrafluoroethylene, vinylidene fluoride, and hexafluoropropylene. The fluorinated elastomer has excellent resistance to oxygen, heat, and swelling by oils and fuels.

Regarding the curing agent shown in Table 1, the polymer chains for NBR composites are mostly linked with the C-S_x_-C bond in the sulfur crosslinked system. The bond energy of C-S is 272 kJ/mol [21,22]. Because the length of the S_x_ chain in the surrounding network is long, the mechanical strength, such as fracture elongation and the elastic modulus, is excellent. Meanwhile, the crosslinking of EPDM polymers with peroxide leads to the formation of C-C bonds between macromolecular chains, which have an energy of 346 kJ/mol higher than that of C-S [21,22]; due to its strong bond energy compared to the sulfur crosslinked system’s, it revealed superior properties in thermal stability, weathering, and in the compression set at elevated temperatures. Therefore, the peroxide crosslinked system is expected to have strong bond energy caused by the dense chain structure resulting in an obstacle for hydrogen permeation.

**Figure 1:**
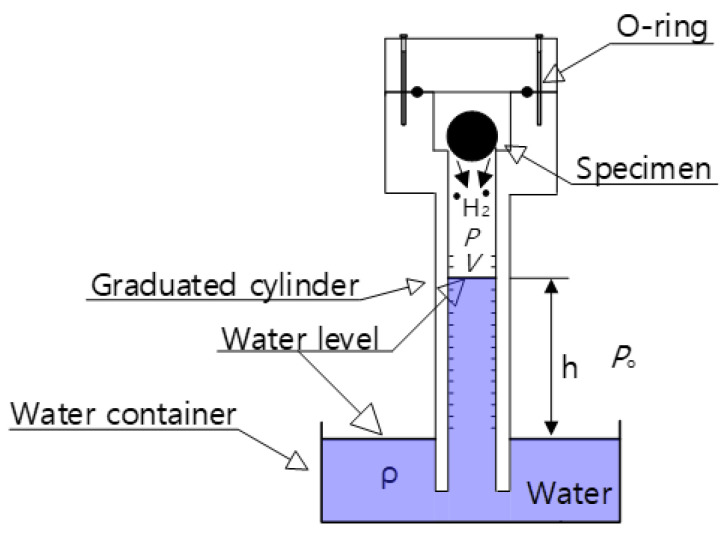
VAT system in which the cylinder is standing upright in distilled water.

The measurements were performed after exposure and subsequent decompression. The polymer specimen was exposed to a fixed pressure of 5.75 MPa for the required residence time. After decompressing the atmosphere, H_2_ gas from the polymer was released. Then, the polymer from the HP chamber was loaded into the graduated cylinder of the VAT system, as shown in Figure 1.

A VAT system measured the released H_2_. A graduated cylinder, immersed partially in a distilled water container, collected and measured the emitted H_2_ gas with an O-ring to prevent a gas leak. The pressure (P) in the cylinder for the H_2_ measurement, shown in Figure 1, is written as [18]

(1) $$P=P_{o}-\rho gh$$

where $P_{o}$ is the atmospheric pressure on the outside of the cylinder, ρ is the density of the distilled water, *g* is gravity, and h is the height of the water level inside the cylinder, measured from the water level in the water container. As shown in Figure 1, the H_2_ gas released from the polymer after decompression lowers the water level of the cylinder, followed by the ideal gas equation, *PV* = *nRT*, where R is the gas constant with 8.20544 × 10^−5^ m^3^·atm/(mol·K). Inside the graduated cylinder filled with gas are *V* and *T*, the upper volume and temperature, respectively, and *n* is the number of H_2_ moles. Thus, we can quantify the amount of emitted H_2_ by measuring the change in the water level (∆V).

The increased moles number (∆n) of H_2_ collected inside the cylinder was obtained by measuring the lowered water level (∆V=A∆h), i.e., volume change (∆V) by H_2_ released from the polymer specimen at the specified *P* and *T* [18]:

(2) $$∆n=\frac{(P_{o}-\rho gh)A∆h}{RT}$$

Here, *A* is the area of the cross-section for the cylinder, and ∆h is the water level lowered by released H_2_. The ∆n of H_2_ was transferred to mass concentration [$C\left(t\right)]$ in the polymer specimen:

(3) $$C\left(t\right)\left[\mathrm{wt}\cdot \mathrm{ppm}\right]=∆n\left[\mathrm{mol}\right]\times \frac{2.016\;\left[\frac{g}{\mathrm{mol}}\right]}{m_{sample}\left[g\right]}$$

where 2.016 [g/mol] is the H_2_ molar mass, and $m_{sample}$ is the specimen mass. Thus, the time-dependent mass content was acquired by measuring the water level change, ∆h, versus the elapsed time.

If we suppose that the sorption and desorption of H_2_ is a diffusion process by Fick law, the released H_2_ concentration, $C_{E}\left(t\right)$, in the sorption process and the remaining H_2_ mass concentration, $C_{R}\left(t\right),$ in the desorption process for a spherical sample are written as Equations (4) and (5), respectively [23,24]:

(4) $$C_{E}\left(t\right)=C_{\infty }\left[1-\frac{6}{\pi^{2}}\overset{\infty }{\underset{n=1}{\sum }}\frac{1}{n^{2}}\exp\left(-\frac{D_{s}n^{2}\pi^{2}t}{a^{2}}\right)\right]$$

(5) $$C_{R}\left(t\right)=C_{0}\frac{6}{\pi^{2}}\overset{\infty }{\underset{n=1}{\sum }}\frac{1}{n^{2}}\exp\left(-\frac{D_{d}n^{2}\pi^{2}t}{a^{2}}\right)$$

$C_{\infty }$ in Equation (4) is the H_2_ mass concentration for a very long duration of time, i.e., the total released mass concentration or H_2_ uptake in the sorption process. $C_{0}$ in Equation (5) is the remaining mass concentration at t = 0 in the desorption process; that is, the total desorption content. a is the radius of the spherical polymer, and $D_{s}\;$and $D_{d}$ are the diffusivity of the sorption process and desorption process, respectively.

In order to analyze the time-varying mass concentration data with the form of a multi-exponential function, a diffusion analysis program to calculate $D_{s}$, $D_{d},$ $C_{\infty }$, and $C_{0}$ in Equations (4) and (5) was utilized [18,25].

## 3. Procedure for Measuring Diffusion Properties in Sorption and Desorption Processes

After the exposure of the specimen in the HP chamber, the sample was removed from the HP chamber and instantly loaded in the top empty volume of the graduated cylinder in the VAT, as shown in Figure 1. The elapsed time after decompression was counted from the moment (*t* = 0) at which the HP chamber’s atmospheric pressure was reduced. Thus, the time lag caused by the sample transfer between decompression and measurement amounted to 5–10 min. The H_2_ emission contents were lost in the transfer time of the specimen. The missing content was measured by extrapolating the simulated line, satisfying the data with the diffusion program. The technique is quite important to obtain precise H_2_ content. The detailed technique is described in the recent research [17].

**Figure 2:**
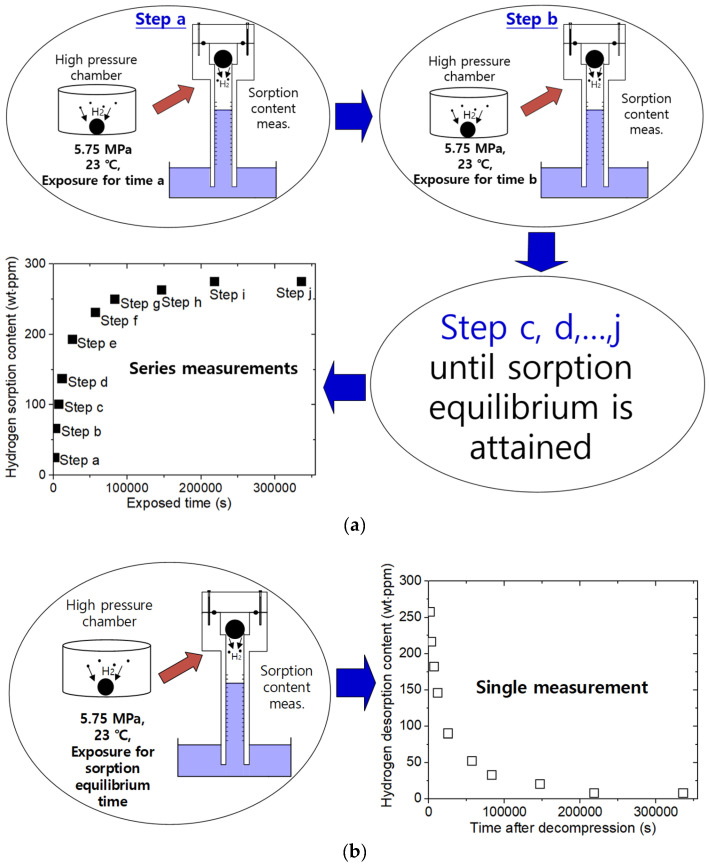
(**a**) A clockwise procedure for measuring sorption properties. (**b**) Procedure for measuring desorption properties. The measurement was performed after loading the cylinder for the specimen’s exposure to the HP chamber.

The procedure for measuring the sorption and desorption properties equated by the H_2_ mass concentration in Equation (3) versus the elapsed time was obtained by VAT after the decompression exposure at a single exposure time, a, as shown in step a of Figure 2a. As a result of this measurement, $c_{0}\left(t=a\right)$ at time a was obtained via Equation (5). As shown in step b of Figure 2a, $c_{0}\left(t=b\right)$ at time b was obtained via Equation (5) after decompression for the exposure at residence time, b. The $c_{0}$ values with varying exposure times (time a, b,…,j in step a, b,…,j, respectively) were collected until H_2_ sorption equilibrium occurred. Thus, the sorption data array was obtained from a series of desorption measurements after subsequent exposure times. From the $c_{0}$ versus the exposure time, shown in Figure 2a, the $C_{\infty }$ and $D_{s}$ of H_2_ were determined by applying the diffusion analysis program based on Equation (4) to the measured results. The sequence for determining the sorption properties required considerable time to complete.

In the desorption process shown in Figure 2b, the H_2_ desorption content and diffusivity were determined from a single measurement after decompression for the exposure of a sufficiently long equilibrium sorption time of the samples in the HP chamber. From the desorption data shown in Figure 2b, $C_{0}\;$and $D_{d}$ were determined by fitting them with Equation (5). Thus, the desorption measurement process could be easily completed in one step and with one sample.

With regard to the two processes, the sorption parameters during pressurization, and the desorption parameters of three types of spherically shaped polymers with different diameters at 5.75 MPa and 296 K, were measured. The sample dimension is directly involved with diffusivity and the hydrogen contents. Thus, we measured the volume variation of the sample during both the compression and decompression by observing the sample in the HP chamber via a transparent sapphire window. The shrinkage during the compression and volume swelling after the decompression were found to be less than 3% at 5.75 MPa for three of the rubbers. The effect was included as a factor of uncertainty evaluation in the previous study [17].

## 4. Results and Discussion

**Figure 3:**
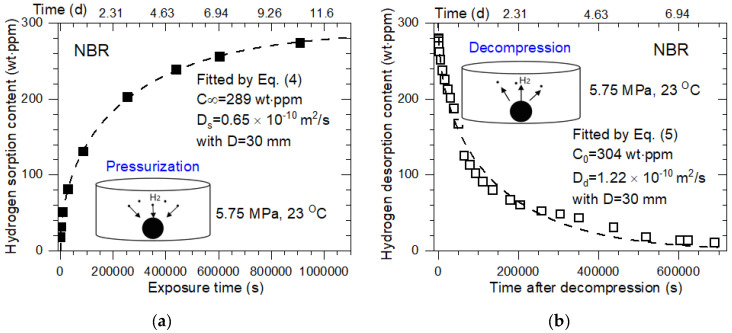

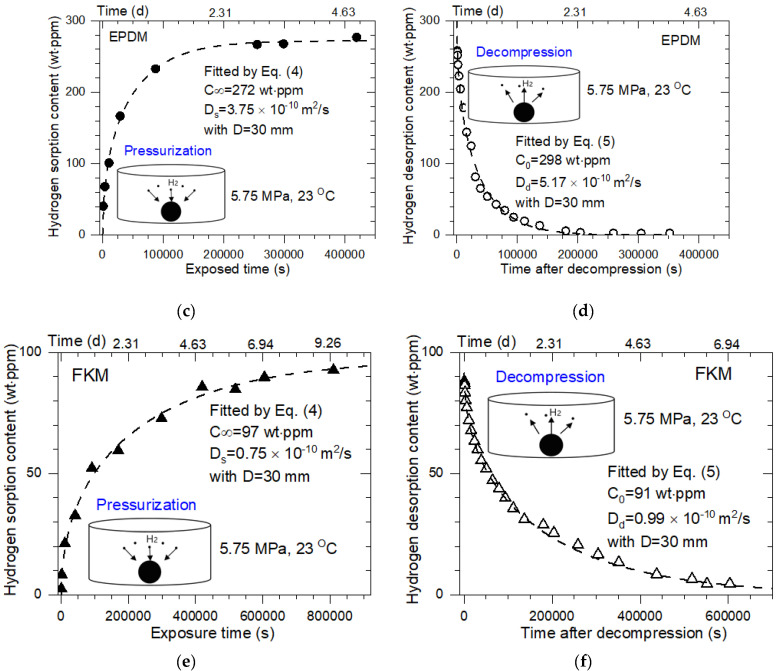
(**a**) H_2_ sorption content versus exposure time and (**b**) H_2_ desorption content versus time after decompression of spherical-shaped NBR samples with diameters of 30 mm. Filled square: sorption data; open square: desorption data; dashed lines: fitted result from Equations (4) and (5). (**c**) H_2_ sorption content and (**d**) H_2_ desorption content for spherically shaped EPDM samples with diameters of 30 mm. Filled circle: sorption data; open circle: desorption data; dashed lines: fitted result from Equations (4) and (5). (**e**) H_2_ sorption content and (**f**) H_2_ desorption content for spherically shaped FKM samples with diameters of 30 mm. Filled triangle: sorption data; open triangle: desorption data; dashed lines: fitted result from Equations (4) and (5).

According to the sorption and desorption procedure shown in Figure 2, the H_2_ diffusion properties in the two processes were measured. Figure 3a–f shows the representative examples of H_2_ sorption and desorption versus the time for spherical-shaped NBR, EPDM, and FKM samples with diameters of 30 mm. The values of $C_{\infty },$ $C_{0}$, *D_s_*, and *D_d_*, analyzed using Equations (4) and (5) with the diffusion analysis program, are presented in Figure 3. Table 2 summarizes the $C_{\infty },$ $C_{0}$, *D_s_*, and *D_d_* of the specimens with other diameters of 10 mm, 15 mm, 20 mm, and 30 mm.

**Figure 4:**
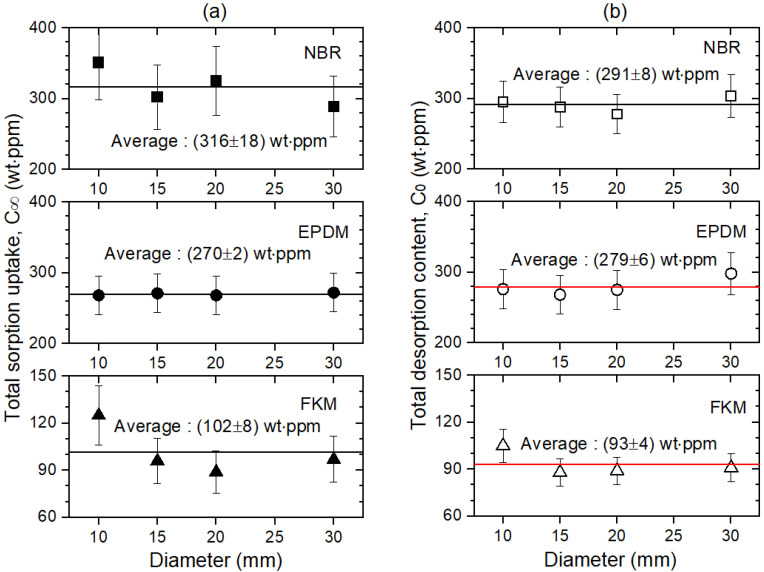
(**a**) Total H_2_ sorption content, $C_{\infty }$, and (**b**) total H_2_ desorption content, $C_{0}$, as a function of the specimen diameter in spherically-shaped NBR, EPDM, and FKM. The horizontal line indicates the average value.

**Figure 5:**
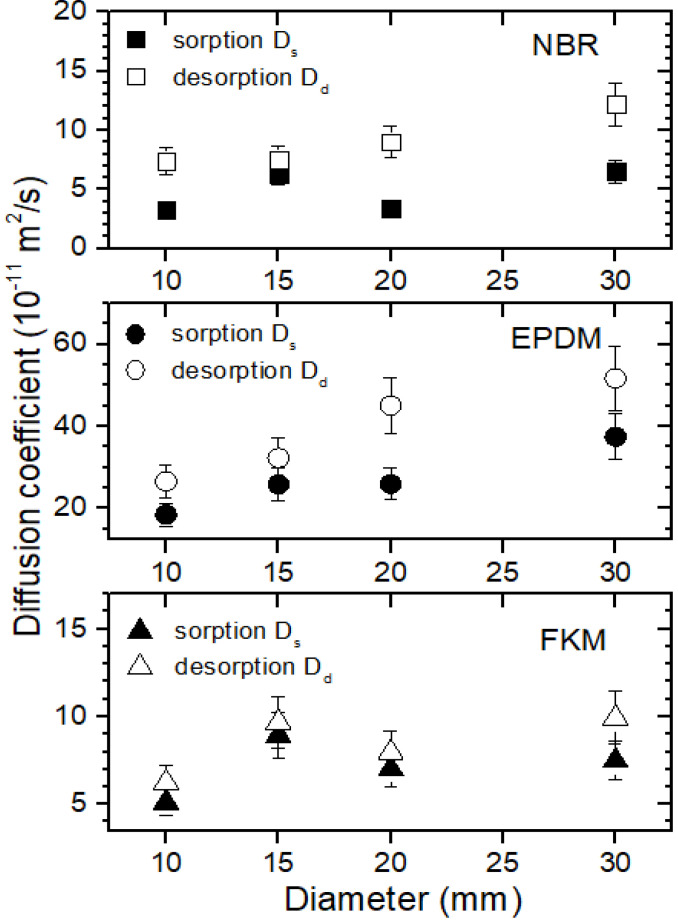
Comparison of the diffusion coefficient ($D_{s}$) in the sorption and diffusivity,$\;D_{d},\;$ in the desorption versus specimen diameter for spherically-shaped NBR, EPDM, and FKM.

For a better view, the H_2_ content and diffusivity investigation results displayed in Table 2 are plotted versus the diameter in Figure 4 and Figure 5, respectively. The general trend, shown in Figure 4, for the three polymers is: both the total sorption content, $C_{\infty },\;$and the total desorption content, $C_{0}$, at the corresponding diameter of each polymer coincide irrespective of the specimen diameter. The average $C_{\infty }$ in each polymer is consistent with the average $C_{0}$ within the uncertainty value. This indicates that the sorption and desorption processes of most H_2_ are reversible, which may be attributed to the physisorption rather than chemisorption by the penetrated H_2_. This result is consistent with previous reports that HP H_2_ exposure does not cause any chemical structure changes in NBR upon nuclear magnetic resonance analysis [26,27]. The reversible sorption phenomenon of hydrogen has been typically observed in the literature [28,29]. In particular, in hydrogen storage materials, the reversibility, in other words, the ability to retain the storage capacity during hydrogen charging and discharging in long-term cycling stability, is a key parameter.

The diffusivity in the desorption process, $D_{d}$, showing the diameter dependency, was faster than $D_{s}$ in the sorption process (Figure 5) for all three polymers. The difference in D observed between the two processes implies that the sorption and desorption processes are different from each other. The fast diffusivity in desorption may be responsible for the increase in H_2_ diffusion due to rapid decompression caused by expanded hydrogen voids, volume expansion, and the chain scission of the polymer. Furthermore, hydrogen penetration causes the scission of the polymer chain. Diffusion takes place in the amorphous region. This phenomenon has also been observed in the literature [15,16].

**Figure 6:**
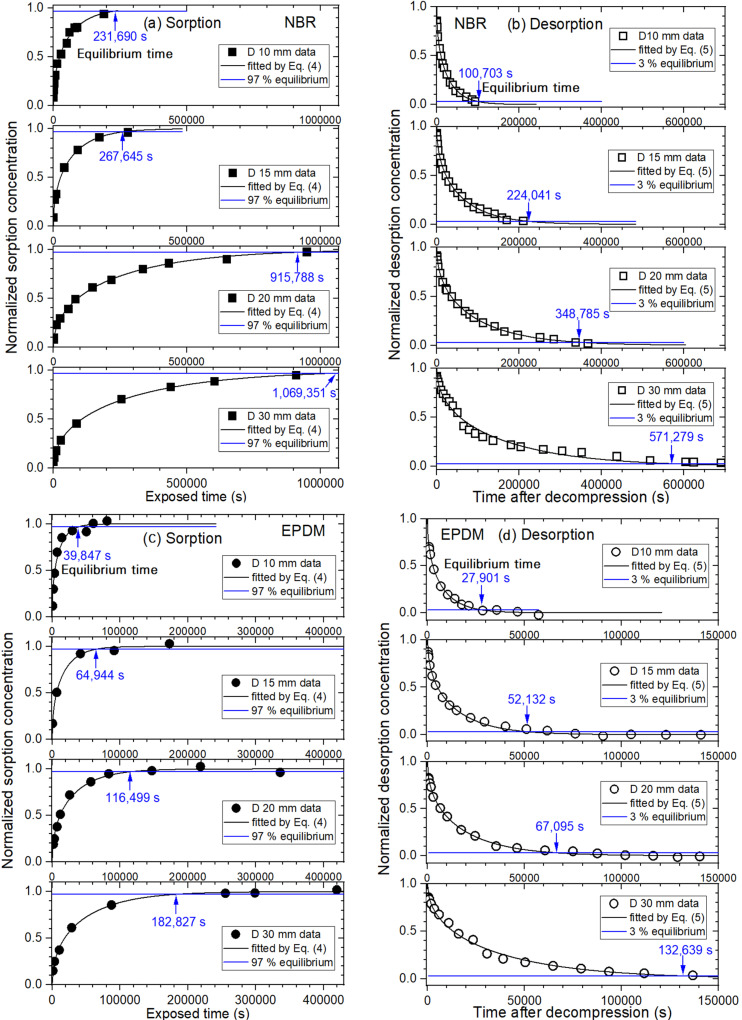

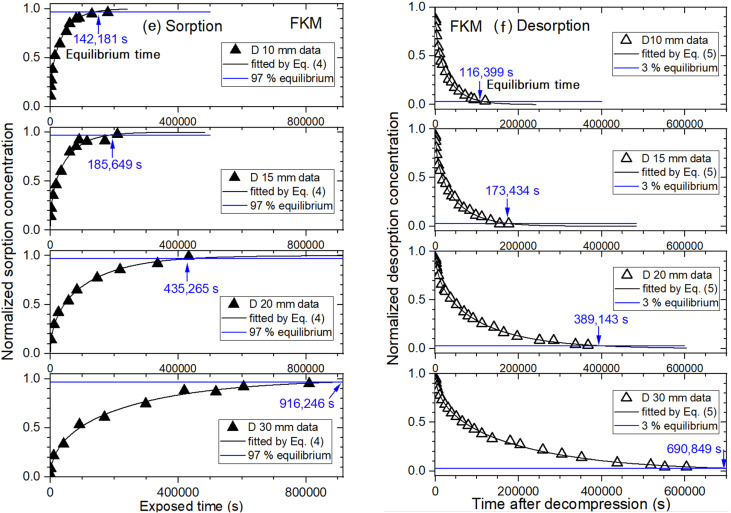
(**a**) Normalized sorption and (**b**) desorption contents versus time for spherically-shaped NBR with diameters of 10 mm, 15 mm, 20 mm, and 30 mm. (**c**) Normalized sorption and (**d**) desorption contents versus time for spherically-shaped EPDM with diameters of 10 mm, 15 mm, 20 mm, and 30 mm. (**e**) Normalized sorption and (**f**) desorption contents versus time for spherically-shaped FKM with diameters of 10 mm, 15 mm, 20 mm, and 30 mm. The arrow indicates the equilibrium time of sorption and desorption.

Because of the multi-exponential form of the sorption and desorption curve with time, the equilibrium time in the two processes is defined as the time at which the H_2_ content reaches 97%, i.e., *C*(*t*) = 0.97 for$\;C_{\infty }$ in Figure 3a and 3%, i.e., *C*(*t*) = 0.03 for $C_{0}$ in Figure 3b. Figure 6 displays the curves of normalized sorption and desorption concentration versus exposure time and time after decompression, respectively. In Figure 6a, the corresponding sorption equilibrium times (blue arrow) obtained for NBR were 231,690 s for 10 mm, 267,645 s for 15 mm, 915,788 s for 20 mm, and 1,069,351 s for 30 mm diameters. The desorption equilibrium times (blue arrow) obtained for the NBR, shown in Figure 6b, were 100,703 s for 10 mm, 224,041 s for 15 mm, 348,785 s for 20 mm, and 571,279 s for 30 mm diameters.

Similar to NBR, Figure 6c,d shows normalized sorption and desorption curves, respectively, for EPDM. The corresponding sorption equilibrium times (blue arrow) obtained for EPDM were 39,847 s for 10 mm, 64,944 s for 15 mm, 116,499 s for 20 mm, and 182,827 s for 30 mm diameters. The desorption equilibrium times (blue arrow) obtained for EPDM were 27,901 s for 10 mm, 52,132 s for 15 mm, 67,095 s for 20 mm, and 132,639 s for 30 mm diameters.

As shown in Figure 6e,f, the corresponding sorption equilibrium times (blue arrow) obtained for FKM were 142,181 s for 10 mm, 185,649 s for 15 mm, 435,265 s for 20 mm, and 916,246 s for 30 mm diameters. The desorption equilibrium times (blue arrow) obtained for the FKM were 116,399 s for 10 mm, 173,434 s for 15 mm, 389,143 s for 20 mm, and 690,849 s for 30 mm diameters.

**Figure 7:**
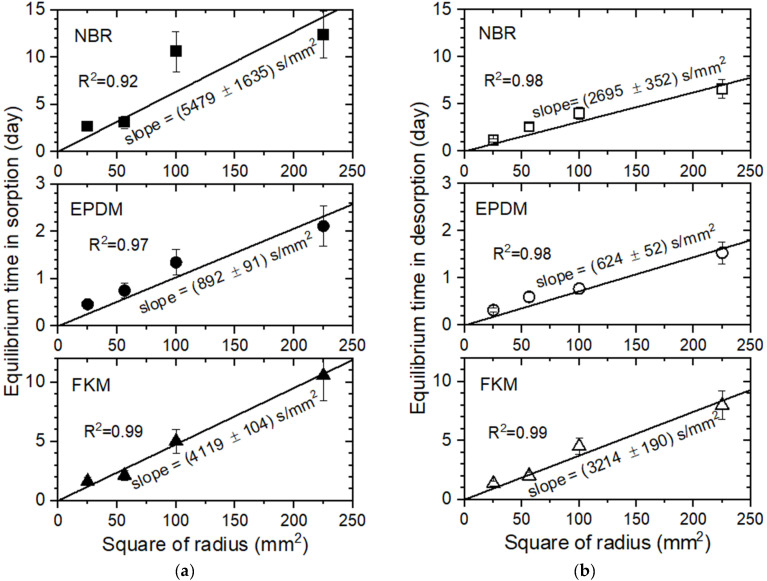

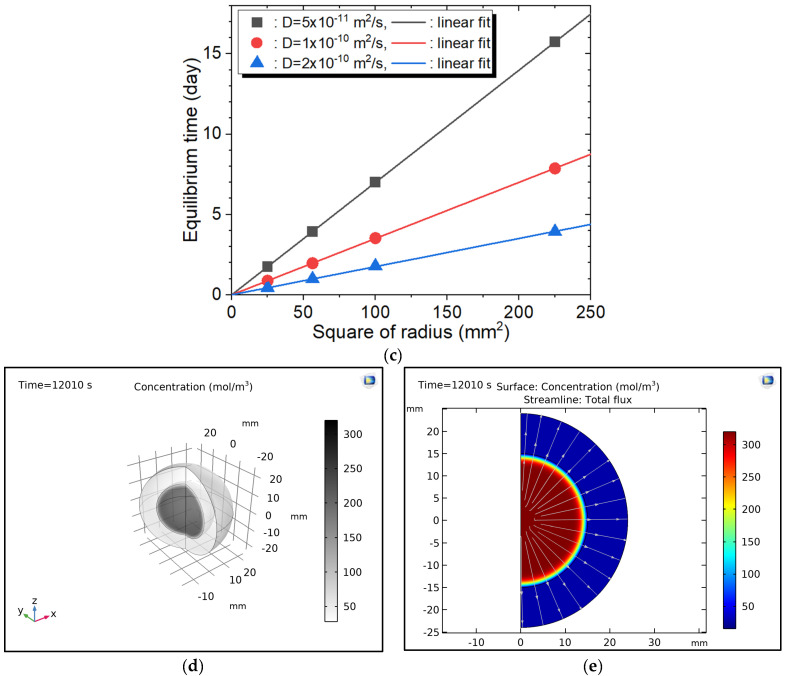
The comparison of the experimental results (**a**,**b**) and COMSOL simulation results, (**a**,**c**–**e**), equilibrium time in sorption and (**b**) equilibrium time in desorption versus the squared radius for NBR, EPDM, and FKM. (**c**) Finite element simulation for the equilibrium time versus the squared radius with diffusivities of 5 × 10^−11^ m^2^/s, 10 × 10^−11^ m^2^/s, and 20 × 10^−11^ m^2^/s; (**d**) a simulation example showing the 3D presentation of the concentration distribution at 12,010 s with a diameter of 30 mm; (**e**) a simulation example showing the concentration distribution at 12,010 s with a diameter of 30 mm.

Figure 7a,b shows the equilibrium time versus the square of the sample radius in the sorption and desorption, respectively, for the three polymers. The experimental observation indicated that the larger the sample diameter was, the longer the time to reach H_2_ uptake saturation. As expected, a linear relationship was found between the saturation time and square of the sample radius, with a well-squared correlation coefficient of R^2^ > 0.92 for the three specimens. The diffusion coefficient in EPDM was faster than that in both the NBR and FKM; this result is attributed to the short equilibrium time, which corresponds to a small slope in the equilibrium time with regard to the square of the radius. The reciprocal slope implies the diffusion coefficient.

According to Equations (4) and (5), the equilibrium time is proportional to the squared radius when the diffusivity is constant, and the diffusion coefficient is a reciprocal slope in the equilibrium time for the squared radius. This is a well-known fact in the case of constant diffusivity. However, because a size-dependent difference in the diffusion coefficient was observed, it is necessary to experimentally confirm whether the linearity between the equilibrium time and squared radius is true or not. Since the experimental results comply with the linearity, the equilibrium time for other diameters is forecasted from the linear correlation without additional measurements.

Furthermore, a numerical simulation using COMSOL was conducted for spherical polymers of different radii with diffusivities of 5 × 10^−11^ m^2^/s, 10 × 10^−11^ m^2^/s, and 20 × 10^−11^ m^2^/s. As shown in the COMSOL simulation results displayed in Figure 7c, the linear dependency between the normalized equilibrium time and squared radius is also shown, which is consistent with the experimental investigation in Figure 7a,b. Figure 7d shows a COMSOL simulation example with the three dimensions of the concentration distribution at 12,010 s with a diameter of 30 mm. Figure 7e is a COMSOL simulation example showing the concentration distribution at 12,010 s with a diameter of 30 mm.

## 5. Conclusions

By utilizing a volumetric analysis technique with a graduated cylinder, we investigated the sorption and desorption parameters of H_2_ in three spherically-shaped polymers for the first time. The H_2_ content, diffusivity, and equilibrium time versus the sample type and diameter were obtained in the sorption and desorption processes. The size dependence was not detected for C_0_ and $C_{\infty }$, while D_s_ and D_d_ were size-dependent.

The reversibility of the H_2_ content measured between the sorption and desorption processes indicated the occurrence of physisorption. The faster diffusivity in the desorption may be attributed to the expanded H_2_ voids, volume expansion, and chain scission of the polymers due to rapid decompression.

The sorption and desorption equilibrium time were sensitive to both the diffusion coefficient and sample radius. Thus, we discovered a method to measure H_2_ sorption saturation before the H_2_ influence of the specimen was tested. The method could be utilized to study the effect of H_2_ and determine the exposure time in H_2_ cycling tests. The time to reach the equilibrium for H_2_ sorption and desorption was observed as linearly proportional to the squared radius, even though diameter-dependent diffusivity was observed. The linearity was also confirmed by the COMSOL simulation. Consequently, with the help of an effective VAT, the equilibrium time of the polymers with different radii could be predicted from the linear correlation without an experimental measurement.

## Figures and Tables

**Table 1:** Functions and chemical compositions in NBR, EPDM, and FKM rubbers.

Function	NBR	EPDM	FKM
Rubber raw material	KNB 35L (100) *	KEP 2320 (100)	VITON 601C (100)
Reinforcing filler	Carbon black, FEF (30)	Carbon black, FEF (30)	Carbon black, MT (25)
Curing agent	Sulfur (2)	Dicumyl peroxide (2)	Calcium dihydroxide (3)
Processing aid	ZnO (3)	ZnO (5)	MgO (4)
Plasticizer	Bis(2-ethylhexyl) adipate (3)	Parraffinic oil (5)	
Accelerators	Tetramethyl thiuram disulfide (1.5)		

* Numbers in parentheses are phr (parts per 100 parts of rubber).

**Table 2:** H_2_ content and diffusivity for the sorption (desorption) process for spherically shaped NBR, EPDM, and FKM at 5.75 MPa and 296 K.

Specimen	$H_{2}\;\mathrm{Content},\;C_{\infty }$ $\left(C_{0})\;[\mathrm{wt}\cdot \mathrm{ppm}\right]$				Diffusion Coefficient, *D_s_* (*D_d_*) [10^−11^ m^2^/s]			
	D 10	D 15	D 20	D 30	D 10	D 15	D 20	D 30
NBR	351 (295)	302 (288)	325 (278)	289 (304)	3.24 (7.40)	6.32 (7.55)	3.36 (9.02)	6.50 (12.2)
EPDM	268 (276)	271 (268)	268 (275)	272 (298)	18.5 (26.6)	25.9 (32.3)	26.0 (45.1)	37.5 (51.7)
FKM	125 (105)	96 (88)	89 (89)	97 (91)	5.11 (6.24)	8.93 (9.68)	7.01 (7.97)	7.49 (9.93)

D 10, D 15, D 20, and D 30 indicate diameters of 10 mm, 15 mm, 20 mm, and 30 mm, respectively, for spherically-shaped polymers.

## Data Availability

The data used to support the findings of this study are available from the corresponding author upon request.
